# Cumulative 10-year performance of endodontically treated teeth with prosthetic restorations of base metal alloy double crowns with friction pins—a retrospective study

**DOI:** 10.1007/s00784-023-05060-9

**Published:** 2023-05-22

**Authors:** Sebastian Hinz, Wolfgang Bömicke, Tobias Bensel

**Affiliations:** 1 Department of Prosthetic Dentistry, University School of Dental Medicine, Martin-Luther-University Halle-Wittenberg, Magdeburger Str. 16, 06112 Halle, Germany; 2 Department of Prosthetic Dentistry, University Hospital Heidelberg, University of Heidelberg, Im Neuenheimer Feld 400, 69120 Heidelberg, Germany; 3 Institute for Research in International Assistance, Akkon University for Human Sciences, Colditzstraße 34-36, 12099 Berlin, Germany

**Keywords:** Double crowns, Friction pins, Root canal treatment, Post and core, Fracture rate, Complication rate

## Abstract

**Objectives:**

This 120-month follow-up study aimed to investigate the complication rate of abutment teeth after endodontic pretreatment with base metal alloy double crowns with friction pins.

**Materials and methods:**

A total of 158 participants (*n* = 71, 44.9% women) aged 62.5 ± 12.7 years with 182 prostheses on 520 abutment teeth (*n* = 459, 88.3% vital) were retrospectively analyzed between 2006 and 2022. Of the endodontically treated abutment teeth, 6.9% (*n* = 36) were additionally treated with post and core reconstructions. Cumulative complication rates were calculated using the Kaplan–Meier estimator and log-rank test. In addition, Cox regression analysis was performed.

**Results:**

The cumulative complication rate at 120 months for the entire set of abutment teeth was 39.6% (confidence interval [CI]: 33.0–46.2). Endodontically treated abutment teeth (33.8%; CI: 19.6–48.0) were found to have a significantly higher cumulative fracture rate than vital teeth (19.9%; CI: 13.9–25.9, *p* < 0.001). Endodontically treated teeth restored with post and core reconstructions (30.4%; CI: 13.2–47.6) showed a nonsignificant lower cumulative fracture rate than that of teeth with root fillings only (41.6%; CI: 16.4–66.8, *p* = 0.463).

**Conclusions:**

Higher 120-month cumulative fracture rates were observed in endodontically treated teeth. Comparable performance was observed in teeth with post and core reconstructions compared to teeth with root fillings only.

**Clinical relevance:**

If endodontically treated teeth are used as abutments for double crowns, the risk of complications from these teeth should be considered when planning treatment and communicating with the patient.

## Introduction

For decades, double crowns have been used successfully to anchor removable partial dentures (RPDs) [1–4]. Double crowns are manufactured in various ways using different materials [5–10]. Particularly, the use of base metal alloy double crowns has increased in recent years [11–13]. The effective life spans of RPDs of 10 years or more have been described under favorable conditions [13–17]. However, biological or mechanical complications may arise during RPD use. Among the biological complications of RPD, fracture of abutment teeth is the most common [2, 18, 19]. The fracture does not necessarily lead to total loss of the impaired abutment tooth or prosthesis. However, abutment tooth fractures always necessitate extensive follow-up treatment. Depending on the study design, fracture rates of up to approximately 15% have been observed [15, 20–24].

Restoring endodontically treated abutment teeth using double crowns causes increased fracture susceptibility of the tooth, which leads to a worse prognosis than vital abutment teeth [2, 13, 15, 25–27]. To stabilize endodontically treated teeth, the use of post and core reconstructions can be considered; however, generally, post and core systems do not strengthen devitalized teeth. Post and core reconstructions are more likely to increase the risk of fatal tooth fractures [28, 29]. These reconstructions should only be used to anchor a core build-up filling in advanced coronal tooth structural loss. Achieving a ferrule design during preparation is important for long-term stabilization of single crowns [30–37]. However, the application of this approach to the anchorage of double crowns in devitalized teeth is not well studied. The rigid connection of the double crowns leads to high stresses in the tooth when loaded in the area of the free-end denture base; however, this could cause problems, especially in severely reduced dentitions [38, 39]. Posts can potentially help stabilize the teeth in this case [20, 40].

This retrospective analysis aimed to compare the fracture rates between vital and endodontically treated double-crowned abutment teeth and those between endodontically treated teeth with and without posts and cores, controlling for other potentially confounding factors such as age, sex, jaw, and abutment teeth number or distribution, over a 120-month period.

Thus, the null hypotheses were: (i) the 120-month fracture rates would be comparable between vital and endodontically treated teeth; (ii) comparable fracture rates would be found between endodontically treated teeth restored with and without posts and cores; (iii) different abutment teeth numbers or distribution would result in comparable fracture rates. In addition, it was hypothesized that potential confounding factors such as age, sex, jaw, and abutment teeth number or distribution would have no effect. Therefore, a 60-month study was conducted [20].

## Materials and methods

### Participants

The study included 158 participants (*n* = 71, 44.9% women); additionally, 182 RPDs were inserted on 520 abutment teeth of these participants. The mean age of participants on the day of placement was 62.6 ± 12.7 years (range, 24.5–87.0). The mean observation period (January 2006 to January 2022) was 67.2 ± 39.7 months (range, 1.4–158.8). All RPDs were provided and followed up at the Department for Prosthodontics at the Martin-Luther-University Halle-Wittenberg.

The protocol of this follow-up study was approved by the Ethics Committee of the Medical Faculty of the Martin Luther University Halle-Wittenberg (registration no. 2016–129) and complies with the Declaration of Helsinki on the Ethical Principles of Medical Research.

### Pretreatment

All participants were thoroughly examined and screened in accordance with the clinical guidelines of the Department of Prosthodontics at Martin Luther University. The necessary conservative or periodontal pretreatment was performed accordingly.

All abutment teeth corresponded to the guidelines of the German Society for Periodontology e.V. (DG PARO) or were brought into this state in advance by systemic periodontal therapy. Specifically, this leads to that all abutment teeth showed pocket depths ≤ 4 mm; horizontal bone loss < 50%; no bleeding on probing (BoP). This results in that only those teeth that were judged to be periodontally healthy were used as abutment teeth. Periodontal therapy was performed during the follow-up period, if necessary.

A temporomandibular disorder (TMD) short screening was carried out as part of the pretreatment as standard.

If indicated, endodontic treatments were performed prior to the definitive treatment with double crown-based RPDs (lateral condensation, ROEKO gutta-percha points, Coltène/Whaledent, Langenau, Germany, AH Plus, DENTSPLY DeTrey, Konstanz, Germany). These root canal fillings were subsequently checked radiographically and had to reach the apical root third to be included in the study.

Endodontically treated teeth with destruction grades I–III (at least two cavity walls preserved) were not reconstructed with a post but were built up with a dual-curing composite (LuxaCore Dual, DMG, Hamburg, Germany). Titanium post and core reconstructions (ER System, Komet Dental, Lemgo, Germany, and LuxaCore Dual, DMG, Hamburg, Germany) were performed on teeth with grade IV destruction (only one cavity wall preserved) [37, 41]. Titanium post and core reconstructions were embedded into the root canal according to the manufacturer’s guidelines using dental cement.

### Inclusion criteria

Only adult participants treated with base metal alloy double crowns with friction pins on all remaining teeth were included. Pregnancy was not an exclusion criterion for this study.

### Exclusion criteria

Participants undergoing radiotherapy for head and neck cancer and those with temporomandibular disorder were excluded. In addition, none of the participants has been treated with an implant-borne restoration. Participants who were suffering from an untreated periodontal disease did not take part in the study. Participants who were suffering from an untreated TMD diagnosis were excluded from the study in advance.

### RPD fabrication

All RPDs were produced in the same dental laboratory (Rübeling + Klar Dental-Labor, Berlin, Germany), according to a standard protocol. Table 1 lists the materials used.

**Table 1:** Composition of materials used for denture fabrication

	Material	Manufacturer	Composition
Sealer	AH Plus	DENTSPLY DeTrey, Konstanz, Germany	Bisphenol A diglycidylether, Bis-[4-(-2,3-epoxypropoxy) phenyl]-methane
Root filling material	ROEKO gutta-percha points	Coltène/Whaledent, Langenau, Germany	Guttapercha, zinc oxide bariumsulfate, and coloring agents
Manufactured root posts	ER System ELO-Stift	Komet Dental, Lemgo, Germany	Titanium
Core built-up material	LuxaCore Dual	DMG, Hamburg, Germany	Acrylate
Impression material	Impregum/Permadyne	3 M ESPE, Neuss, Germany	Polyether
Secondary crowns Dentures frameworks	Okta-M	SAE, Bremerhaven, Germany	Cobalt 60–66%, chromium 27–32%, and molybdenum 5–7%
Denture base material	FuturaGen	Schütz Dental GmbH, Rosbach v.d.H., Germany	Polymethylmethacrylat, dibenzoylperoxid, and methyl-methacrylat
Prosthetic teeth	Primodent	Polident d.d., Dental Products Industry, Volčja Draga, Slovenija	Polymethylmethacrylate, dimethacrylates, and pigments
Luting agent	Hoffmann’s CEMENT normal setting;	Hoffmann Dental Manufaktur, Berlin, Germany	Zinc oxide and phosphoric acid
Indicator silicone	Fit Checker TM Advanced	GC Corporation, Tokyo, Japan	VPES-silicone, vinylpolyether

Tooth preparations were performed using rotary diamond instruments (Komet Dental, Gebr. Brasseler GmbH & Co. KG, Lemgo, Germany). The controlled circular tooth substance removal was 1.0–1.5 mm, and the retentive preparation was performed with a preparation angle of approximately 4–6° [42]. For all teeth, care was taken to ensure that the preparation chamfer ended at least 2 mm below the reconstruction (“ferrule design”) [30–37]. All tooth impressions were made using a polyether material (Impregum, Permadyne, 3 M ESPE, Neuss, Germany). In addition, all primary crowns were produced with a tapered angle of 2° from a cobalt-chromium-molybdenum alloy (Okta-C SAE DENTAL VERTRIEBS GMBH, Bremerhaven, Germany). Clinically, the internal fit of the primary crowns was checked with low-viscosity silicone (Fit CheckerTM Advanced, GC EUROPE N.V., Leuven, Belgium), and the position of the primary crowns on the abutments was transferred into a new master cast using a polyether border molding pick-up impression.

Subsequently, the denture frameworks were manufactured, and the friction pins were incorporated. A passive fit was achieved by the spark erosion process, in which an insertion groove (0°) was introduced into an approximal surface of the primary crown. Furthermore, the corresponding friction pin (Ø = 0.7–0.9 mm) was fixed in the secondary crown using laser welding [43–48]. Centric relation, occlusion, framework design, and aesthetics were checked during the subsequent overall try-in of the dentures.

Special attention was paid to the periodontally hygienic design of the dentures. Definitive placement of all primary crowns was performed with zinc oxide phosphate cement (Hoffmann’s CEMENT, normal setting, Hoffmann Dental Manufaktur, Berlin, Germany). All treatment steps were performed by calibrated practitioners. Finally, all participants were provided with detailed instructions on the correct handling and care of the dentures after completion.

### Data collection

Retrospective data collection was based on participant charts and anonymized. The following data were collected: age; sex; date of insertion of the denture; date of the last dental check-up; supplied jaw; denture classification according to Steffel [49]; position of the abutment teeth according to the Fédération Dentaire Internationale (FDI) scheme; vitality of the abutment teeth; number of double crowns per denture; number of lost retaining elements; number of relining; number of activations of the friction pins; date and reason for loss of function of the dentures and abutment teeth. The sample size was not determined for this explorative study. All patients who fit the profile (treatment with double-crown-retained RPD with friction pins) and received prosthodontic treatment between 2006 and 2022 were included in the study. Only patients with follow-up clinical data were included; accordingly, three additional patients who had received an RPD of interest during the indicated period were excluded because they never returned for follow-up.

### Follow-up

For participants with this type of prosthesis, the follow-up interval was set at 6 months. Further follow-up visits were scheduled according to the participants’ individual circumstances. The 6-month routine follow-up examinations were performed by trained and calibrated practitioners of the Department of Prosthetic Dentistry of the University School of Dental Medicine of the Martin-Luther-University Halle-Wittenberg.

### Statistical analysis

The participants were subdivided into two groups depending on the number of remaining abutment teeth: (i) ˃ three teeth, non-severely reduced dentition (NSRD); (ii) ≤ three teeth, severely reduced dentition (SRD). In the SRD group, the distribution of abutment teeth was subdivided according to the Steffel classification [13, 49] for further evaluation: class A, one remaining tooth with punctual support; class B, two remaining teeth with linear sagittal support; class C/D, two remaining teeth with linear transversal/diagonal support; and class E, three remaining teeth with triangular support.

Observation time was defined as the time from insertion of the final prosthesis to the day of complication onset. Complications were defined as tooth loosening due to periodontitis, abutment tooth fracture, caries, endodontic disease, or primary crown loss. Cumulative complication and fracture rates at 120 months were calculated using the Kaplan–Meier method. The confidence interval (CI) was set at 95% [50].

The influence of variables such as age, sex, jaw, dentition status (SRD vs. NSRD), abutment tooth type, abutment teeth vitality, and endodontic treatment on the long-term success of abutment teeth was investigated using the log-rank test and/or Cox regression. Significance was set at *α* = 0.05 for all analyses. All calculations were performed using IBM SPSS 28 statistical software (IBM Corp., Armonk, USA).

## Results

### Participants

Between 2006 and 2022, a total of 158 patients (*n* = 71; 44.9% women; mean age, 62.6 ± 12.7 years [range, 24.5–87.0]) received dentures with base metal alloy double crowns with friction pins. A total of 182 dentures (*n* = 92, 50.5% maxillary and *n* = 90, 49.5% mandibular) were inserted on 520 abutment teeth. Of these abutment teeth, 61 were endodontically treated before the placement of double crowns (Table 2).

**Table 2:** Participants and abutment teeth characteristics

Abutment teeth			
Variable		Number	Percentage (%)
Sex	Men	296	56.9
	Women	224	43.1
Jaw	Maxilla	262	50.4
	Mandible	258	49.6
Dentition status	NSRD	206	39.6
	SRD	314	60.4
	Class A	30	5.8
	Class B	84	16.2
	Class C/D	68	13.0
	Class E	132	25.4
Endodontic status	Vital	459	88.3
	Root filling	25	4.8
	Root filling + post	36	6.9
Tooth type	Incisors	79	15.2
	Canines	220	42.3
	Premolars	159	30.6
	Molars	62	11.9
	Total	520	100.0

*SRD*, severely reduced dentition; *NSRD*, not severely reduced dentition

Of the 61 endodontically treated abutment teeth, the degree of destruction of 25 teeth was classified as class I–III and received only root canal filling. Meanwhile, 36 endodontically treated abutment teeth were classified as class IV and received a post in addition to root canal fillings.

The SRD and NSRD denture groups included 314 and 206 abutment teeth, respectively.

All RPDs were supported periodontally by natural abutment teeth and mucosally in the area of the residual alveolar ridge. Of the 182 RPDs examined, 48 (26.08%) dentures were relined during the observation period: 23 in the maxilla and 25 in the mandible. Mean observation time was 67.2 ± 39.7 months (range, 1.4–158.8). Sixty-five percent of all patients adhered to recommendations for biannual recall.

### Abutment tooth complications

During the study period, 75 (14.0%) abutment teeth were fractured, of which 58 (42 vital, 8 endodontically treated, and 8 endodontically treated with a post) were immediately extracted. However, 17 fractured abutment teeth could be reconstructed, and their function was retained. In addition to fractures, other complications occurred in the abutment teeth, such as periodontal damage (*n* = 24, 4.6%), endodontic disease (*n* = 21, 4.0%), and caries (*n* = 13, 2.5%) (Table 3).

**Table 3:** Complication of abutment teeth over examination time

Complications	Endodontic status			Total (%)
	Vital (%)	Root filling (%)	Root filling + post (%)	
Without complications	349 (76.0)	14 (56.0)	23 (63.8)	386 (74.2)
Loosening due to periodontitis	23 (5.0)	1 (4.0)	0 (0.0)	24 (4.6)
Fracture	57 (12.4)	8 (32.0)	10 (27.8)	75 (14.4)
Caries	12 (2.6)	0 (0.0)	1 (2.8)	13 (2.5)
Endodontic disease	17 (3.8)	2 (8.0)	2 (5.6)	21 (4.1)
Loss of primary crown	1 (0.2)	0 (0.0)	0 (0.0)	1 (0.2)
Total	459 (100.0)	25 (100.0)	36 (100.0)	520 (100.0)

### Root canal treatment after double crown restoration

Of the 520 abutment teeth, 50 (9.6%) required endodontic treatment during the wearing period of the double-crown prosthesis. Of these, three (0.6%) were secondary endodontic treatments (revisions).

The mean time to endodontic treatment was 44.62 ± 4.997 months (range, 1.58–144.23), and the median time was 35.08 months.

### Cumulative complication rate determined using Kaplan–Meier method—total number of abutment teeth

**Fig. 1:**
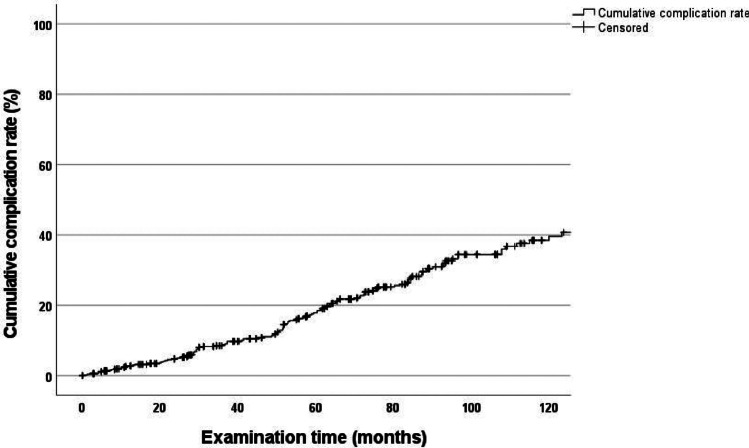
Cumulative complication rate of all abutment teeth

**Fig. 2:**
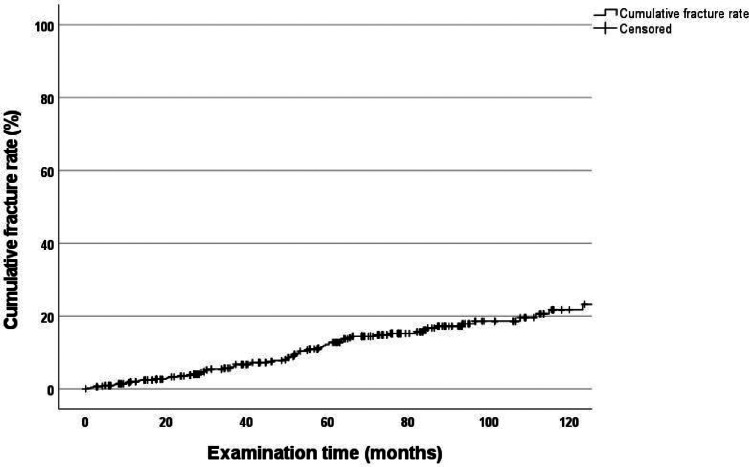
Cumulative fracture rate of all abutment teeth

The 120-month cumulative complication rate for all abutment teeth was 39.6% (CI: 33.0–46.2) for all types of complications (Fig. 1). The cumulative fracture rate for all abutment teeth was 21.8% (CI: 16.2–27.4) for this period (Fig. 2).

### Cumulative fracture rate determined using Kaplan–Meier method—vital vs. endodontically treated

**Fig. 3:**
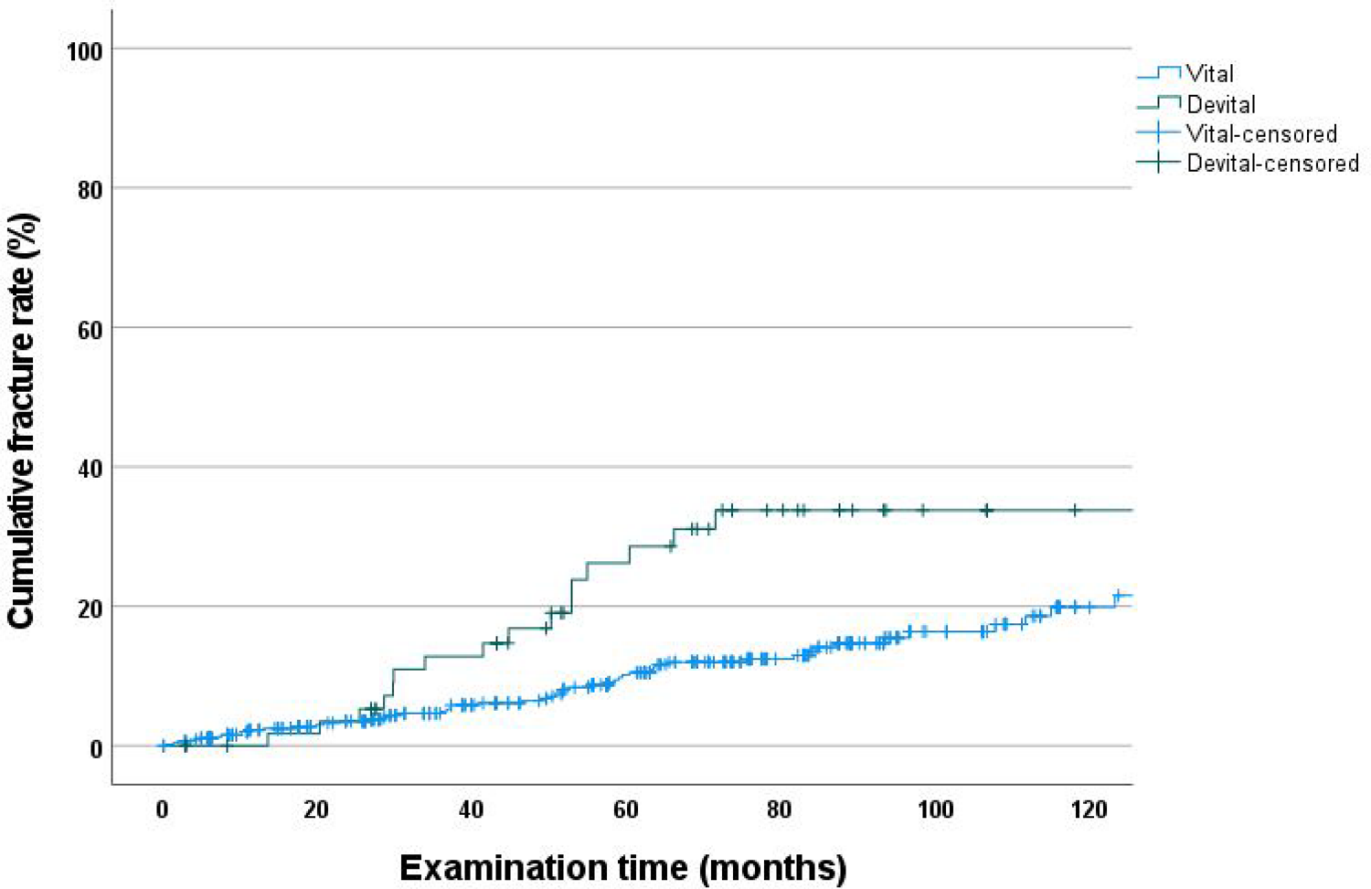
Cumulative fracture rate as a function of vitality

At 120 months, the cumulative fracture rate for endodontically treated abutment teeth (33.8%; CI: 19.6–48.0) was higher than that of vital abutment teeth (19.9%; CI: 13.9–25.9; log-rank test, *p* < 0.001) (Fig. 3).

### Cumulative fracture rate according to Kaplan–Meier—endodontically treated without post vs. endodontically treated with post

**Fig. 4:**
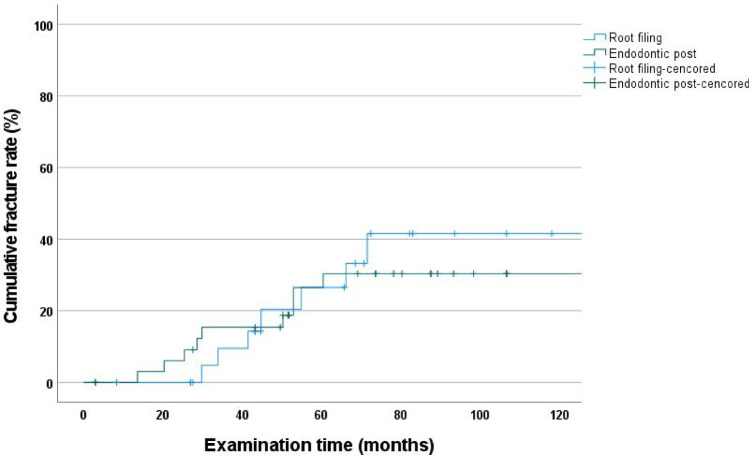
Cumulative fracture rate as a function of a post and core treatment before crowning

At 120 months, the cumulative fracture rates for endodontically treated teeth without posts (41.6%; CI: 16.4–66.8) and those with posts (30.4%; CI: 13.2–47.6) were not significantly different (log-rank test, *p* = 0.463) (Fig. 4).

### Cumulative fracture rate according to Kaplan–Meier—SRD vs. NSRD

**Fig. 5:**
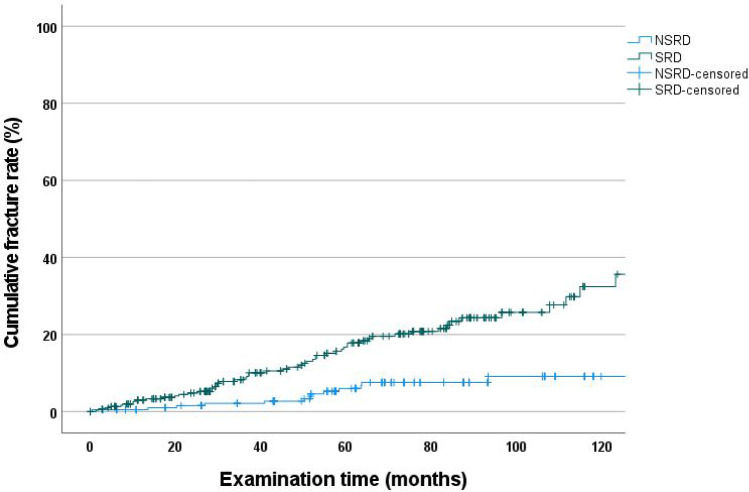
Cumulative fracture rate as a function of the number of abutment teeth

At 120 months, the cumulative fracture rates for abutment teeth (9.1%; CI: 3.9–14.3%) in NSRD dentures were significantly lower than those for SRD dentures (32.4%; CI: 22.6–42.2; *p* < 0.001) (Fig. 5).

### Cumulative fracture rate as a function of severely reduced dentition—Steffel classification (A–E)

**Fig. 6:**
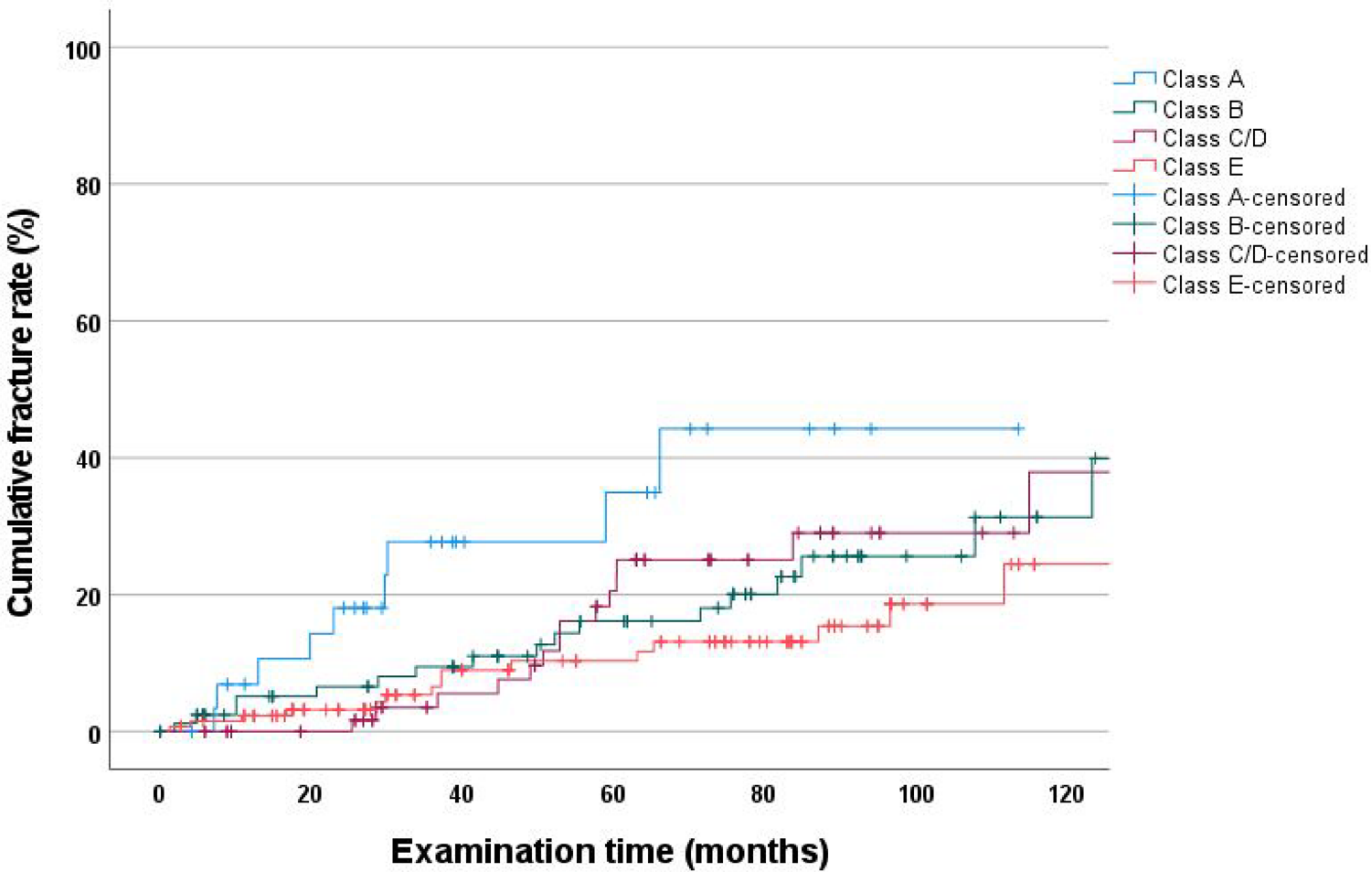
Cumulative fracture rate as a function of severely reduced dentition

Statistically significant differences were found between Steffel classes A–E (*p* = 0.015). Class A had the highest cumulative fracture rate (44.2%; CI: 19.0–69.4); however, a follow-up period of 120 months was not reached in this class (only 113 months). Class C/D (37.9%; CI: 17.1–58.7) had the second highest cumulative fracture rate at 120 months, followed by class B (31.3%; CI: 15.5–47.1). Class E (22.5%; CI: 7.7–37.3) had the lowest rate (Fig. 6).

### Cumulative endodontic treatment rate

**Fig. 7:**
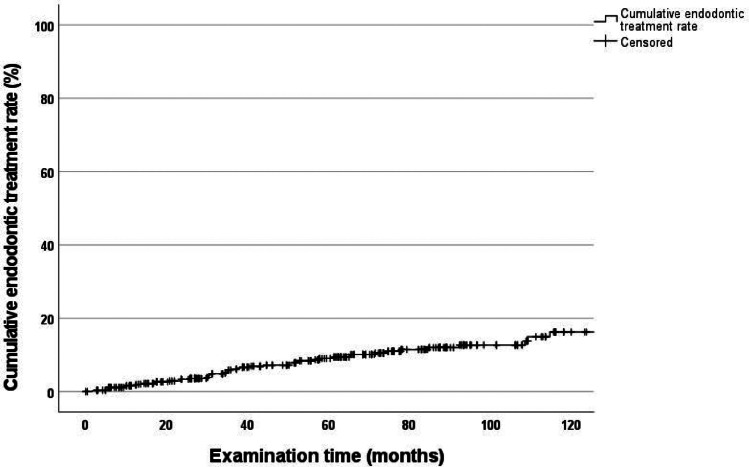
Cumulative endodontic treatment rate

After 120 months, the cumulative endodontic treatment rate for all abutment teeth was 16.3% (CI: 10.9–21.7) (Fig. 7).

### Cox regression

For abutment tooth fracture, a multivariate analysis was performed with the following variables: age, sex, jaw, endodontic status, dentition status (number of abutment teeth, i.e., SRD or NSRD), Steffel class (A–E), and tooth type. Estimated hazard ratios (HRs) and 95% CIs are shown in Table 4.

**Table 4:** Hazard ratios of the different variables included in multivariate analysis

Abutment teeth				
Variable		Hazard ratio	95% CI	*P*-value
Age		1.043	1.021 to 1.065	< 0.001
Sex	Men vs. women	1.335	0.835 to 2.135	0.228
Jaw	Maxilla vs. mandible	0.783	0.487 to 1.260	0.313
Endodontic status	Vital vs. endodontically treated	3.035	1.735 to 5.308	< 0.001
Endodontic treated	Reference vital			< 0.001
	Root filling	3.159	1.497 to 6.667	0.003
	Root filling + post	2.345	1.194 to 4.606	0.013
Dentition status	NSRD vs. SRD	3.107	1.786 to 5.405	< 0.001
SRD	Reference NSRD			< 0.001
	Class A	7.239	3.200 to 16.372	< 0.001
	Class B	2.754	1.418 to 5.351	0.003
	Class C/D	2.946	1.459 to 5.950	0.003
	Class E	1.948	0.988 to 3.843	0.054
Tooth type	Reference molars			0.077
	Incisors	1.531	0.531 to 4.414	0.431
	Canines	2.582	1.017 to 6.557	0.046
	Premolars	1.559	0.582 to 4.176	0.377

*RPD*, removable partial denture; *SRD*, severely reduced dentition; *NSRD*, not severely reduced dentition; *Class A*, one remaining tooth with punctual support; *Class B*, two remaining teeth with linear sagittal support; *Class C/D*, two remaining teeth with linear transversal/diagonal support; *Class E*, three remaining teeth with triangular support

Age, endodontic status, dentition status, and Steffel class were found to have a significant effect on the fracture rate of abutment teeth.

## Discussion

The present follow-up study aimed to investigate the complication rate of abutment teeth after treatment with base metal alloy double crowns with friction posts within a 120-month observation period, with consideration of the distribution of abutment teeth and their endodontic pretreatments.

This study had several null hypotheses. First, the 120-month fracture rates would be comparable for vital and endodontically treated teeth and for endodontically treated teeth with and without posts and cores. Second, we hypothesized the same to be true for different numbers and distributions of abutment teeth. Finally, potential confounding factors such as age, sex, jaw, and the abutment teeth number and distribution would have no effect.

In the present study, the cohort size, as well as the number of abutment teeth, was sufficiently large compared to that of previous studies [10, 13, 15].

Cumulative complication rate of abutment teeth at 120 months was 39.6%, which was higher than that reported in other studies [2–4]. However, most comparable studies reported only short study periods. Compared with a previous 60-month study, we found a 15.5% increase in cumulative complication rate [20].

The present study found a 14.4% fracture rate, which was higher than those in comparable studies [2, 15, 21, 22]. Compared with a 60-month study, our study showed a 3.5% increase in fracture rate [20]. The need for endodontic treatment after restoration of abutment teeth with double crowns was 10.2% at 120 months. This result corresponds to an increase of approximately 3% compared to the results of a previous study with a study period of 60 months [20]. The cumulative fracture rate of endodontically treated abutment teeth (33.8%) was significantly higher than that of vital abutment teeth (19.9%) at 120 months. Comparable results have been shown in other studies [1–3, 20, 22, 23, 27, 51].

In the present study, endodontically treated abutment teeth with post and core build-ups had a lower fracture rate (30.4%) at 120 months than endodontically treated abutment teeth with root fillings only (41.6%), although endodontically treated abutment teeth had less initial damage. However, the difference was not statistically significant, suggesting that post-and-core treatments do not increase fracture rates. Rather, fracture rates tended to decrease. This trend was observed in a previous 60-month study [20]. Furthermore, these results were consistent with those of other studies, where post and core abutments showed better results [18, 40]. Thus, the null hypothesis that post-and-core restored abutment teeth have similar fracture rates as root-filled-only abutment teeth was accepted.

Cox regression analysis showed that the risk of failure was approximately 200% higher for endodontically treated abutment teeth than for vital abutment teeth (*p* < 0.001, HR = 3.035). We compared vital teeth with abutment teeth that received post and core reconstructions of 130% (*p* = 0.013, HR = 2.345) and abutment teeth that received root-filled reconstructions of only 210% (*p* = 0.003, HR = 3.159); we found that abutment teeth with post and core reconstructions performed better (Table 4).

This 120-month follow-up study suggests that post and core reconstructions might be recommended as pretreatment for root-filled teeth prior to the use of double crowns, which was also suggested in a previous 60-month study [20] and is consistent with that of other studies [18, 40].

Direct post and cores are frequently used for the reconstruction of abutment teeth for RPD [36]. The literature does not contain universally valid statements on the benefits of post and core systems. Studies examining single-tooth restorations showed high success rates, even in severely destroyed teeth that were built up exclusively with composites [52, 53]. In contrast, studies in which endodontically treated teeth were restored with post and core as RPD abutments showed better long-term results than those restored with composite alone [18, 20, 40].

Only a direct titanium post and core were used in the present study because of the degree of destruction of the abutment teeth. This type of post shows good results in the long-term restoration of teeth, achieving similar or even better success rates than fiber posts [54–58]. In contrast, a previous 60-month study included a small number of cast post and core abutments, which were excluded from the present study design [20]. For all tooth preparations, care was taken to achieve a “ferrule design” with a height of at least 2 mm above the chamfer margin line. This is known to demonstrate a considerable influence on long-term success, irrespective of the post and core system. The advantages of a “ferrule design” have been demonstrated [30–32, 59, 60]. In addition, we can assume that free-end situations (Kennedy classes I and II) can present with excessive stress on the terminal abutment teeth if these dentures anchored with double crowns are loaded [38, 39]. All remaining teeth were used as abutment teeth and received double crowns. All secondary crowns were fitted with a friction pin. The advantage of these friction pins is that the retention of the denture can be adjusted individually. In addition, in case of a wearing period over several years, it is possible to activate the prosthesis and restore retention without replacing parts [13].

Notably, in the present 120-month follow-up study, more than 60% of the abutment teeth were part of a severely reduced dentition. Particularly in the case of abutment teeth with severely reduced dentition, high vertical and horizontal loads on the remaining abutment teeth are expected owing to the special gap situation. Correspondingly, the cumulative fracture rate of abutment teeth was significantly lower in NSRD dentures (9.1%) than in SRD dentures (32.4%) at 120 months. This finding is consistent with other studies that have shown that long-term survival of dentures was associated with the number of abutment teeth available [15, 20, 23, 40].

Regarding the results of the individual Steffel classes, the lowest cumulative fracture rates were observed in class E (22.5%). This finding demonstrates the importance of the number of abutment teeth and is consistent with that of other studies [20, 23].

Cox regression analysis showed that the number and distribution of abutment teeth significantly influenced fracture rates (Table 4). Abutment teeth in the SRD group had an approximately 200% higher risk of fracture than those in the NSRD group. In addition, individual Steffel classes differed significantly, with class A having a 600% higher risk of abutment tooth fracture than the teeth in the NSRD. This is consistent with the results of a 60-month study [20]. Thus, the greater the number of abutment teeth, the lower the overall fracture rate.

In addition to vitality and number of teeth, Cox regression analysis showed that age had a major influence on the fracture rate of abutment teeth. The fracture rate increased correspondingly with patient age (4.3% per year, *p* < 0.001). This result is consistent with the results of a 60-month study [20]. Possible reasons for this may be declining manual dexterity and associated difficulties in daily oral hygiene in elderly patients [61, 62].

The reliability of the results must be discussed critically. This study had several limitations. First, because the data were analyzed retrospectively using patient records, only documented events were included in our analysis. Factors such as the restoration of the opposing jaw, unless it was also a denture with double crowns with spark-eroded friction pins, were not considered in this study. Different prostheses are expected to produce different chewing forces. This confounding factor could not be accounted for in our analysis. Second, the periodontal condition of the abutment teeth was not specifically considered; however, only those teeth that were judged to be periodontally healthy were used as abutment teeth. Periodontal therapy was performed during the follow-up period, if necessary. Third, although 6-month check-ups are standard, some patients were noncompliant. This applied to about 35% of patients. Thus, if more regular check-ups and follow-up treatments had been performed, the fracture rate of abutment teeth might have improved. Regular recall may increase the success rate of dentures [63]. Fourth, all RPDs were produced using the same procedures in a single dental laboratory; however, dental treatment was performed by multiple dentists with different clinical experiences. Fifth, effectiveness of the post and core system used in this study has been demonstrated, but whether the system is optimal for the reconstruction of abutment teeth under DCP dentures remains to be proven. Sixth, the distribution of abutment teeth (SRD and NSRD) was not similar in both groups, which may have led to bias in the results. Ideally, both study groups should be of equal size. However, it is not possible to define this in advance in the context of a retrospective clinical follow-up. Overall, the sample size in both groups appeared to be sufficient compared to that in other studies [20, 23].

Seventh, posttreatment and fitting procedures were performed by different dentists. Due to national health care regulations, if there are only three or fewer remaining teeth, the costs for double crowns are largely covered by the insurance. For this reason, this treatment is used very often in severely reduced dentitions. Nonetheless, all of them adhered to the standards agreed to by the department where the study was conducted, which may effectively reflect the reality of clinical practice.

The fracture rate of abutment teeth increased compared to the result of the 60-month follow-up examination but was within the generally expected range. Generally, if abutment teeth are fractured, they can often be rebuilt. Alternatively, the abutment tooth can continue to be used with a root post cap and a ball abutment. Overall, the use of double crowns with spark-eroded friction pins can be advocated in compliance with the influencing factors shown.

## Conclusion

Within the limitations of this retrospective study, endodontically treated abutment teeth showed higher fracture rates than vital teeth after 10 years. The abutment teeth treated with a post and core restoration showed a lower probability of fracture than teeth that were exclusively root canal-filled and built up with composite. However, this difference was not statistically significant. Thus, it could be stated that posts do not have a negative impact on fracture susceptibility but rather have a positive effect on double-crowned teeth. In addition, the number of abutment teeth may be a relevant factor for the fracture risk of abutment teeth.
